# A retrospective feedback analysis of objective structured clinical examination performance of undergraduate medical students

**DOI:** 10.12688/mep.20456.1

**Published:** 2024-10-24

**Authors:** Akram Alsahafi, Micheál Newell, Thomas Kropmans

**Affiliations:** 1 College of Medicine, Nursing and Health Sciences – School of Medicin, University of Galway, Galway, County Galway, Ireland; 2 Department of Medical Education, College of Medicine, Taif University, Taif, Makkah Province, 11099 - 21944, Saudi Arabia

**Keywords:** OSCE, medical education, feedback quality, written feedback, assessment

## Abstract

**Introduction:**

Feedback is an essential component of medical education, enhancing the quality of students' knowledge and skills. However, providing effective feedback, particularly in clinical skills assessments like Objective Structured Clinical Examinations [OSCEs], often poses challenges. This study aimed to evaluate the content of OSCE feedback given to undergraduate medical students over five years.

**Methods:**

A retrospective analysis of 1034 anonymised medical students' OSCE performance was conducted, focusing on written feedback. The written feedback data were randomly selected from OSCE sessions, collected from university assessment records and anonymised for ethical considerations. R software was used to identify the most frequently repeated words in the examiners’ feedback text, and word cloud charts were created to visualise the responses.

**Results:**

Word clouds generated from the top 200 most frequently used terms provided visual insights into common descriptive words in feedback comments. The most frequently repeated word over five years was "good," indicative of potentially non-specific feedback.

**Discussion:**

The high frequency of non-specific terms like "good" suggests a need for more specific, constructive feedback. However, such generic terms can offer some positive reinforcement, more than they may be needed to foster significant improvement. As previously proposed in the literature, adopting structured feedback forms may facilitate the delivery of more specific, actionable feedback.

**Conclusion:**

This study emphasises the importance of providing specific, actionable feedback in medical education to facilitate meaningful student development. As medical education continues to evolve, refining feedback processes is crucial for effectively guiding students' growth and skill enhancement. Using structured feedback forms can be a beneficial strategy for improving feedback quality.

## Practical points

•   The study highlights the importance of structured and specific feedback in improving the quality of OSCE assessments, thereby enhancing medical education.

•   Text mining revealed "good" as the most frequent term descriptor in OSCE feedback, indicating a lack of specificity and actionability.

•   Word clouds showed common term descriptors in feedback, highlighting focus areas and gaps in current practices.

## Introduction

Providing feedback is an important part of learning and teaching in medical education ( Bing-You *et al.*, 2017; Ende, 1983; Hattie & Timperley, 2007; Shrivastava & Shrivastava, 2020a). Feedback helps learners bridge their existing gaps and move forward towards the competencies and skills expected of them ( Gupta *et al.*, 2021). It provides essential information that plays a critical role in eliminating uncertainties related to course content and desired competencies, thereby guiding learners on their educational journey ( Shrivastava & Shrivastava, 2020b). Effective feedback in medical education is crucial for promoting learning among students ( Shrivastava & Shrivastava, 2020b). It encourages reflection on practice, helping students to understand their strengths and areas for improvement ( Bakke *et al.*, 2020). Additionally, structured and actionable feedback can significantly enhance students' clinical skills and patient care practices ( Lai *et al.*, 2020).

In the context of Objective Structured Clinical Examinations (OSCEs), feedback from examiners is particularly valuable as it provides detailed insights into students' clinical competencies and areas for improvement ( John *et al.*, 2021). OSCEs are structured assessments where students demonstrate their clinical skills through various stations, and examiners document their observations and provide feedback ( John *et al.*, 2021). Analysing this feedback can discover patterns and trends that inform teaching practices and student learning strategies ( Gilkes *et al.*, 2022). Text analysis, which involves extracting meaningful information from textual data, has become an increasingly popular approach for examining written feedback ( Khanbhai *et al.*, 2021). By leveraging tools like R and R Studio, educators can systematically analyse large volumes of feedback, identifying common themes and specific areas where students frequently struggle ( Hynninen *et al.*, 2020). This process enhances the understanding of student performance and guides the development of targeted educational interventions ( Sharma & Jain, 2021).

Text analysis in educational research has shown promising results in enhancing feedback mechanisms ( Ferreira-Mello *et al.*, 2019). Text analysis allows for systematically examining qualitative data, providing insights that might not be immediately apparent through manual review ( Castleberry & Nolen, 2018). In medical education, particularly in OSCEs, this technique can evaluate the quality and content of examiners' feedback ( Maimone *et al.*, 2023). Researchers can perform sophisticated analyses, such as sentiment analysis, frequency analysis, and topic modelling, using R and R Studio to understand feedback patterns better ( Welbers *et al.*, 2017). These analyses can reveal how feedback is distributed across different competencies, identify recurrent student issues, and highlight areas where feedback might lack specificity or constructiveness ( Chary *et al.*, 2019). Moreover, the results from text analysis can inform the training of examiners, ensuring that feedback is comprehensive and delivered in a manner that maximises student learning and development ( Chary *et al.*, 2019).

This study aims to use text analysis techniques in R and R Studio to systematically evaluate and improve the quality of written feedback provided by examiners in OSCEs. By identifying the key descriptors and visualising the most repeated words in feedback, this study seeks to enhance the feedback process, ultimately supporting better learning outcomes for medical students.

## Methods

### Study design

In this retrospective study, records of 1,034 different undergraduate medical students' OSCE performance across five separate cohorts were analysed. The clinical skill assessments of OSCE covered a range of anonymised stations of OSCEs, including medical history, physical examinations, or clinical procedures, such as CNS, respiratory, abdominal, and OBGYN exams. As our study is retrospective and based on the examiners' feedback stored in the university’s records, the waiver of consent was granted as part of the ethical approval by the University Ethics Committee of the University of Galway, which oversees all studies involving human participants. All extended data, including the feedback text, and data processing codes, have been uploaded to our repository on Zenodo alongside the underlying data.

### OSCE, scores, written feedback

The OSCE is a valid and reliable assessment tool for clinical skills assessment in medical and health sciences education. During the OSCE, examiners assess student performance and input the students' observed marks on score sheets. They can also provide their professional opinion on students' performance using the following categories of the Global Rating Scale (GRS) (Fail, Borderline Fail, Borderline Pass, Good, or Excellent). In addition, there is a general comments section for examiners to give written feedback.

### Data collection and analysis

This project utilised OSCE performance data extracted from the Objective Structured Clinical Examination (OSCE) digital assessment platform (Qpercom Observe; https://www.qpercom.com). The data was collected by the University of Galway. According to university procedures, the examiners' feedback is collected during the exams. The data extraction date (access to the university records) was April 5, 2021. Data collection consisted of OSCE scores, Global Rating Scale results, and examiner feedback from a random selection of OSCE examinations from a single institution over a five year period. All data was anonymised.

**Figure 1.**
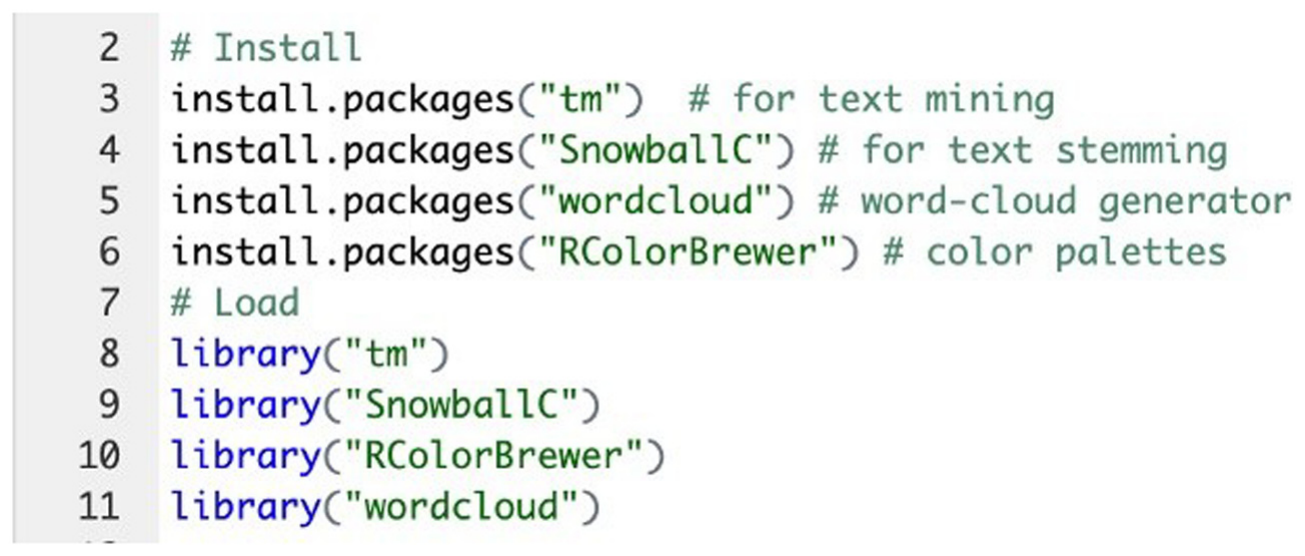
R Code implementation: Initial Data Loading Step.

Text mining methods highlighted the most frequently used keywords in a paragraph of text. A series of word clouds, called text clouds or tag clouds, were created to represent text data visually. The text mining package and the word cloud generator package in R software were used to create the word clouds. The OSCE results data was saved as a text file, and the following R codes were generated ( Figure 1):

### Text mining overview

Text mining, also known as text analytics, derives meaningful information from natural language text. It involves various techniques such as text preprocessing, tokenisation, and feature extraction to analyse and interpret textual data. In this study, we utilised text mining to analyse examiners' feedback in the OSCE context. This approach allows for the systematic identification of patterns, themes, and sentiments within the feedback, providing insights that can enhance the quality of feedback provided to medical students ( Kumar, 2015; Zong *et al.*, 2021)

### Tools and software

The text mining analysis used R, a programming language and software environment for statistical computing and graphics ( Li *et al.*, 2017). R Studio, an integrated development environment (IDE) for R, facilitated coding and data visualisation ( Li *et al.*, 2017). These tools were selected for their robust text mining packages, extensive community support, and capabilities for data manipulation and visualisation.

### Text preprocessing

**Figure 2.**
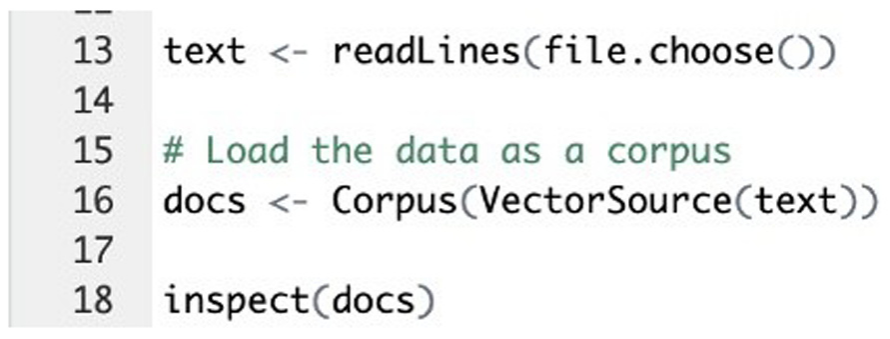
R code implementation: Document import and inspection step.

**Figure 3.**
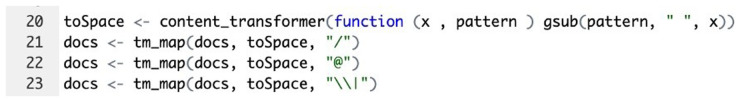
R code implementation: Text transformation and special characters removal step.

**Figure 4.**
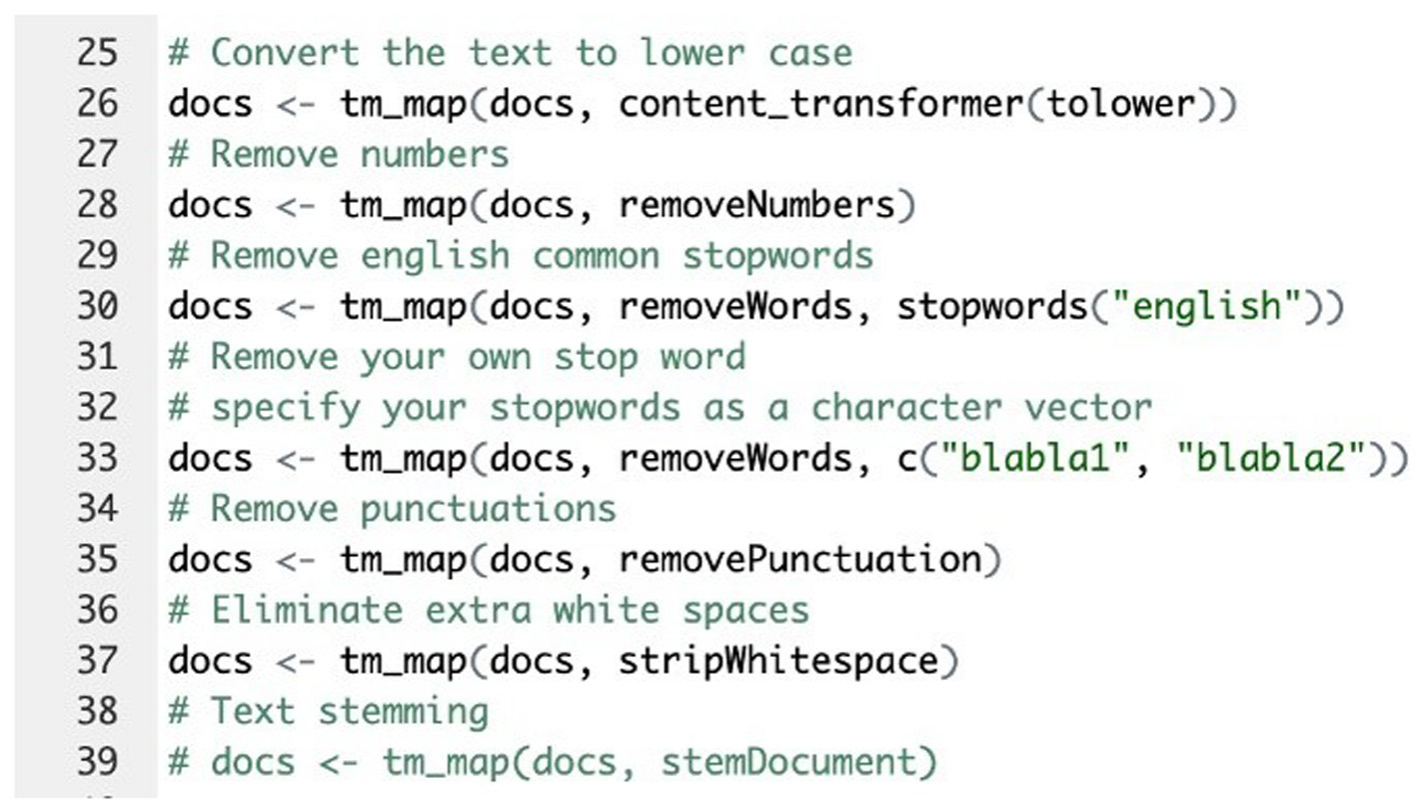
R code implementation: Text cleaning - removing unnecessary spaces, numbers, and converting to lowercase.

The initial step involved loading the feedback data into R using the Corpus() function from the tm package ( Figure 1). After loading the text data, it was inspected using the inspect() function to ensure accuracy and completeness ( Figure 2). Text transformation was then performed using the tm_map() function to replace special characters, such as "/", "@", and "|", with spaces ( Figure 3). Finally, text cleaning was conducted by removing unnecessary whitespace, converting the text to lowercase, removing common stop words, and eliminating numbers and punctuation using the tm_map() function ( Figure 4).

### Term-document matrix

**Figure 5.**
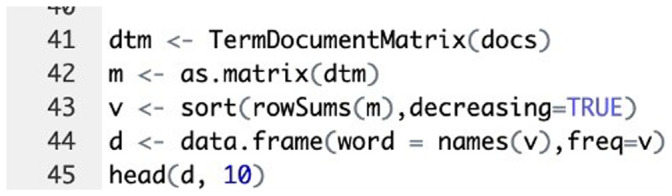
R code implementation: Term document matrix creation for word frequency analysis.

A term-document matrix (TDM) was created to quantify the frequency of words in the feedback data. The TDM represents the occurrence of terms in the documents, which is essential for further analysis and visualisation ( Figure 5).

### Word cloud generation

**Figure 6.**
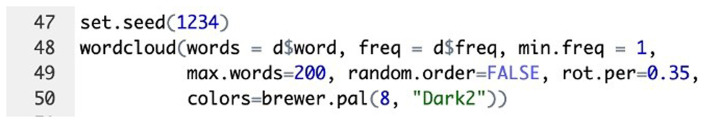
R code implementation: Word cloud generation for visualisation of word frequencies.

A word cloud was generated to visualise the most frequent terms in the feedback data. The word cloud package was utilised for this purpose. The word cloud provides a visual representation where the size of each word indicates its frequency in the text. The following steps were taken to generate the word cloud ( Figure 6):

1. **Term frequency calculation:** The frequency of each term was calculated from the TDM.
2. **Word cloud creation:** The word cloud function was used to create the word cloud, with customisation options for colour and layout provided by the R Colour Brewer package.

### Ethical considerations

As our study is retrospective and based on the examiners' feedback stored in the university's records, the waiver of consent was granted as part of the ethical approval by the University Ethics Committee of the University of Galway, which oversees all studies involving human participants. All extended data, including the feedback text, and data processing codes, have been uploaded to our repository on Zenodo alongside the underlying data. The University Ethics Committee of the University of Galway granted ethical approval for the study on December 2nd, 2020, with the Ethical Committee Application Reference Number 2020.12.019. The study adheres to the principles outlined in the Declaration of Helsinki.

## Results

**Figure 7.**
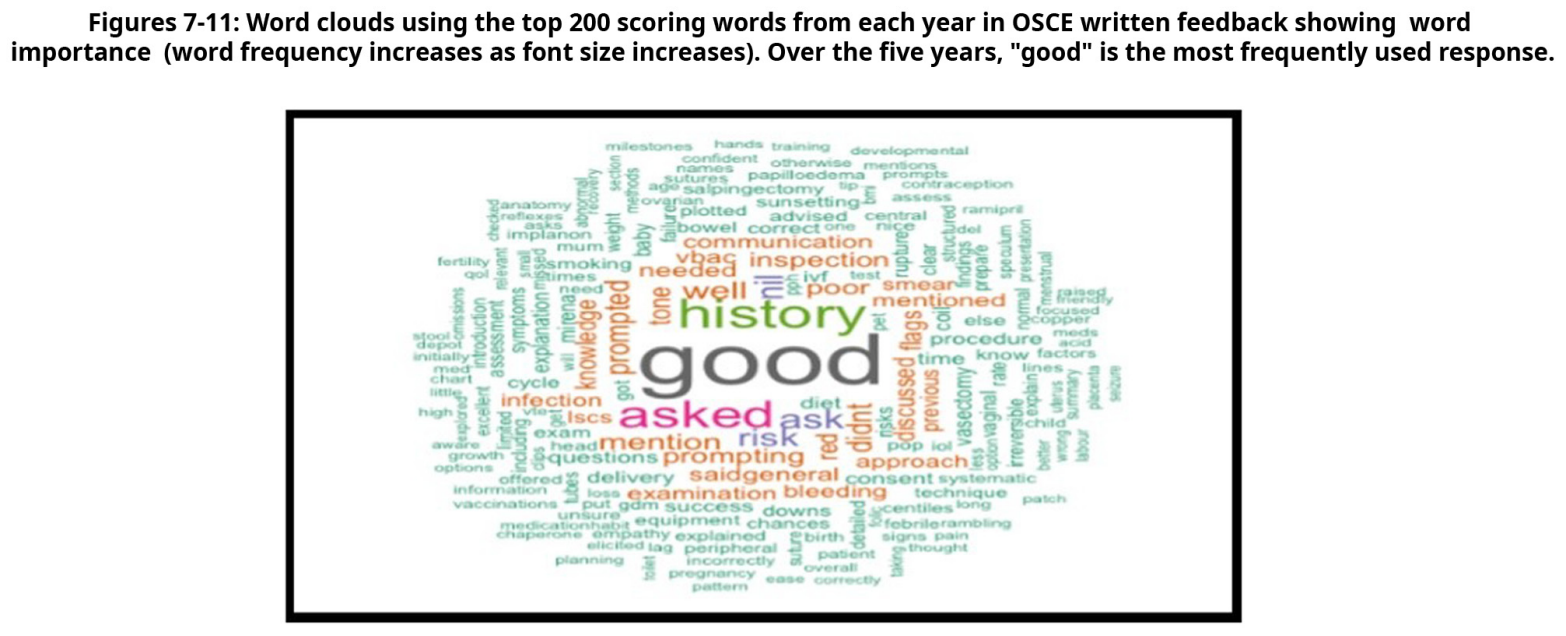
Top 200 scoring words cloud in year 1.

The analysis spanned five cohorts of a single academic year, containing the feedback data of 1034 anonymised undergraduate medical students. Examiners’ feedback from randomly selected OSCE sessions was meticulously explored and analysed. The top 200 most recurrent terms in the examiners' written feedback were extracted to identify descriptors. These terms were visually represented through word clouds in Figures 7 through Figure 11, respectively.

**Figure 8.**
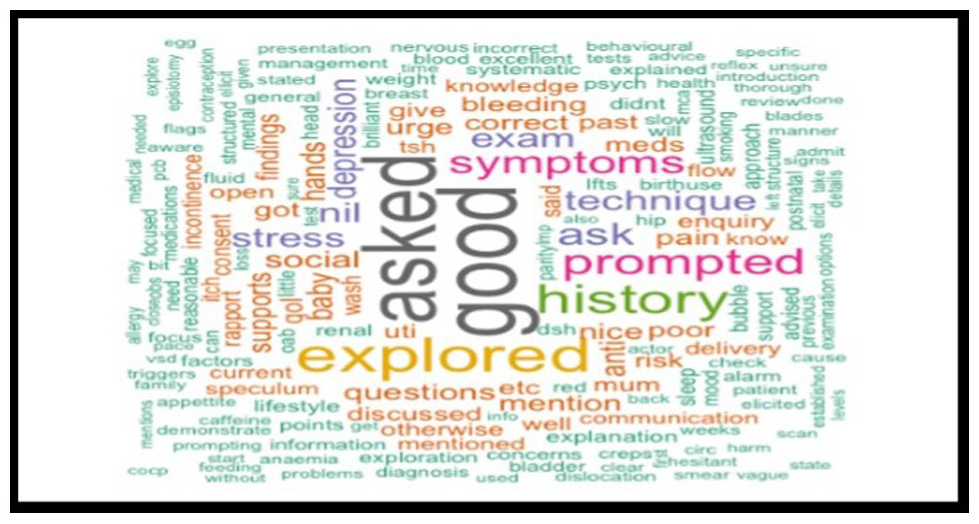
Top 200 scoring words cloud in year 2.

**Figure 9.**
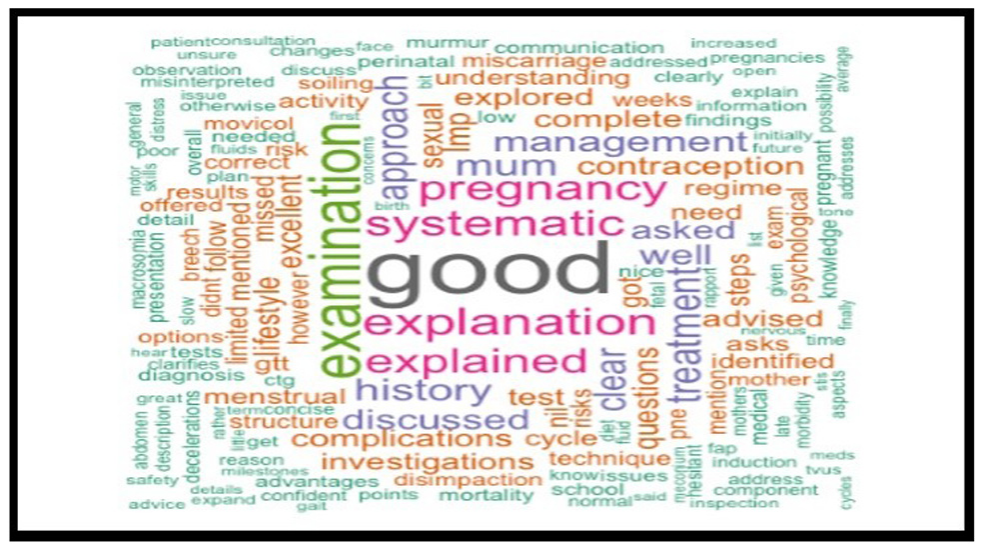
Top 200 scoring words cloud in year 3.

**Figure 10.**
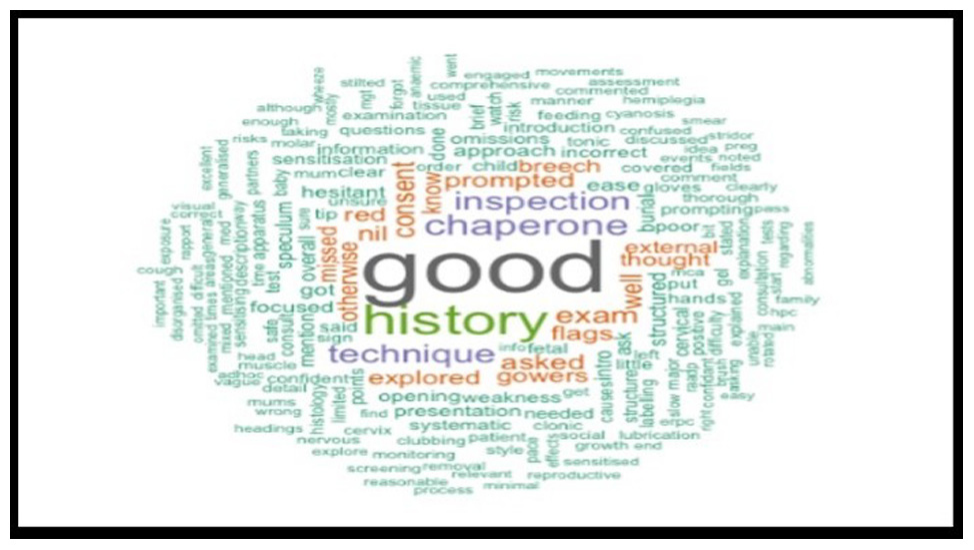
Top 200 scoring words cloud in year 4.

**Figure 11.**
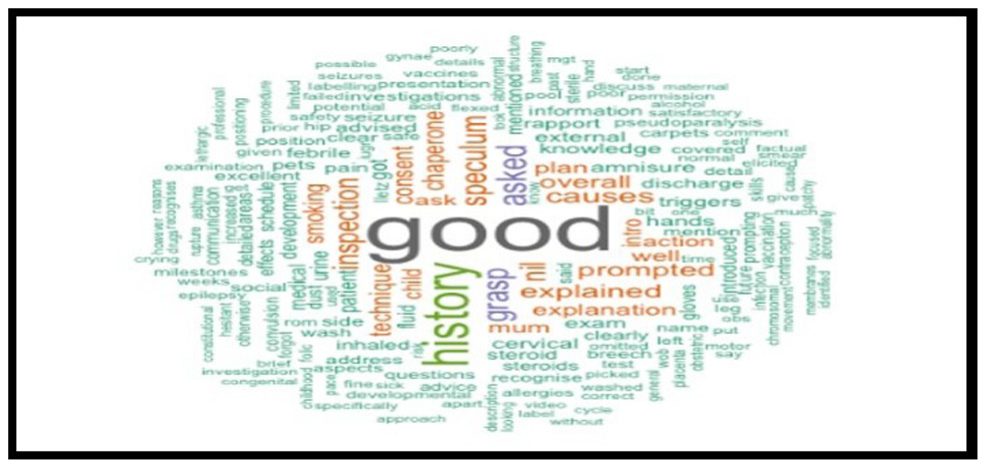
Top 200 scoring words cloud in year 5.

A word's prevalence is visually conveyed through its size in word clouds. The more frequently it appears in the source text, the more pronounced it becomes within the cloud. This visualisation method is a straightforward and intuitive approach to deciphering qualitative data and pinpointing key themes. Word clouds offer a rapid understanding of the main focal points within a text by providing a quick snapshot of the data's core aspects. This easy-to-digest representation facilitates a more efficient grasp of information, allowing major themes or recurring words to stand out significantly. Incorporating word clouds into our analysis allowed us to depict graphically the frequency and prominence of the term "good" in the examiner's feedback. This visualisation indicates the word's prevalence, illustrating its dominance within the evaluative commentary provided during the examination period.

## Discussion

This study's primary objective was to analyse the written feedback presented during OSCE examination sessions, particularly emphasising the most frequently used words in examiner comments. This analysis could illuminate the efficacy of the feedback provided to medical students and shed light on potential areas for improvement in the feedback process ( Bajaj *et al.*, 2018; Bing-You *et al.*, 2017; Haffling *et al.*, 2011).

The text mining analysis and word cloud visualisation results highlight that particular descriptive terms frequently emerge in the feedback comment ( Carr, 2006). Some of these descriptive words highlight areas where medical students consistently perform well, while others highlight areas where improvement is required. However, nebulous feedback such as "good" may not be enough to facilitate meaningful progress. To maximise the effectiveness of feedback, it is necessary to adhere to specific guidelines that encourage actionable, appropriate, and constructive responses ( Alsahafi *et al.*, 2022; Burgess *et al.*, 2020; Gigante *et al.*, 2011).

Although comments such as "good" may provide some positive reinforcement and motivation, more specific and actionable feedback is generally more beneficial ( Ende, 1983; Hattie & Timperley, 2007). Such feedback gives the recipient a thorough understanding of their performance and offers detailed guidance on sustaining or enhancing their skills.

Implementing structured feedback forms, as proposed by Wardman *et al.* (2018), is a beneficial method for enhancing the quality of written feedback in OSCEs. These written feedback forms use predetermined statements customised to each OSCE station to enable examiners to provide more specific, balanced, and constructive feedback. Feedback should describe the gap in student learning and observe behavioural actions in the exams ( Alsahafi *et al.*, 2022; Wardman *et al.*, 2018).

In light of advancing technological interventions, the potential of embedding artificial intelligence AI in the OSCE feedback process emerges as a compelling consideration ( Han *et al.*, 2019). As feedback mechanisms evolve, (AI) may have a greater role in the examination process. By seamlessly integrating these capabilities within the existing OSCE feedback system, there's an opportunity for real-time, detailed, and objective feedback generation ( Zhang *et al.*, 2023). Focusing on the meticulous evaluation of scoresheet items, scores, and GRS, AI can intelligently synthesise this data to produce comprehensive, consistent, and specific student feedback. The advantage extends beyond the precision; rapid response times ensure that students receive feedback immediately relevant to their performance ( Han *et al.*, 2019; Zhang *et al.*, 2023). Such timely insights can guide students towards more focused learning, capitalising on the benefits of immediate reinforcement. Furthermore, with AI's adaptive learning capabilities, the system could continually refine itself, offering even more personalised and accurate feedback, modernising the feedback mechanism and ensuring students benefit from actionable insights.

This study's limitations are that it focused on a singular institution and a subset of undergraduate medical students. Therefore, the findings may only apply to some medical students or institutions ( Cushing *et al.*, 2011). In addition, the text mining analysis provided only the frequency of certain words, which may not wholly capture the nuances of the provided feedback ( Carr, 2006).

In conclusion, generic terms such as "good" may not give recipients the necessary information to comprehend their performance or influence their development. Adopting a more comprehensive approach to feedback, as described above, may enhance medical students' learning and development over time ( Ende, 1983; Hattie & Timperley, 2007).

## Conclusion

This study demonstrates the value of analysing written feedback during OSCE examination sessions. However, the prevalence of vague and generic comments, such as "good," highlights the need for more actionable and constructive feedback that may enhance learning development among undergraduate medical students.

Using structured feedback forms, exams may provide more specific, pertinent, and actionable feedback addressing students' strengths and weaknesses. This method assists undergraduate medical students in comprehending their performance and provides direction for enhancing their skills and knowledge.

## Ethics and consent

As our study is retrospective and based on the examiners' feedback, the waiver of consent was granted as part of the ethical approval by the University Ethics Committee of the University of Galway, which oversees all studies involving human participants. All extended data, including the feedback text, and data processing codes, have been uploaded to our repository on Zenodo alongside the underlying data. The University Ethics Committee granted ethical approval for the study on December 2nd, 2020, with the Ethical Committee Application Reference Number 2020.12.019. The study adheres to the principles outlined in the Declaration of Helsinki. The primary data was collected by the University of Galway during the OSCE exams, following the university's protocols and procedures.

## Data Availability

Zenodo: A Retrospective Feedback Analysis of Objective Structured Clinical Examination Performance of Undergraduate Medical Students. https://doi.org/10.5281/zenodo.11096861 ( Alsahafi *et al.*, 2024) This project contains the following underlying data:
- Feedback Text (The raw text data was collected from participants and used to generate the word clouds. This includes all examiners' feedback and comments.) Data are available under the terms of the Creative Commons Attribution 4.0 International license (CC-BY 4.0). Analysis code available from: https://github.com/Akram-09/OSCE_Cloud_Study_Codes.git Archived analysis code at time of publication: https://doi.org/10.5281/zenodo.12593752 ( Akram-09, 2024) License: CC-BY 4.0

## References

[ref-1] AKRAM-09: Akram-09/OSCE_Cloud_Study_Codesv_ 1.0.0. Zenodo. 2024. 10.5281/zenodo.12593752

[ref-2] Alsahafi A Ling DLX Newell M : A systematic review of effective quality feedback measurement tools used in clinical skills assessment. *MedEdPublish (2016).* 2022;12:11. 10.12688/mep.18940.1 37435429 PMC10331851

[ref-3] Alsahafi A Newell M Kropmans T : A retrospective feedback analysis of objective structured clinical examination performance of undergraduate medical students.[Dataset]. Zenondo. 2024. 10.5281/zenodo.11096861 PMC11615435 39635542

[ref-4] Bajaj JK Kaur K Arora R : Introduction of feedback for better learning. *J Clin Diagn Res.* 2018. 10.7860/JCDR/2018/36744.12402

[ref-5] Bakke BM Sheu L Hauer KE : Fostering a feedback mindset: a qualitative exploration of medical students’ feedback experiences with longitudinal coaches. *Acad Med.* 2020;95(7):1057–1065. 10.1097/ACM.0000000000003012 32576764

[ref-6] Bing-You R Hayes V Varaklis K : Feedback for learners in medical education: what is known? a scoping review. *Acad Med.* 2017;92(9):1346–1354. 10.1097/ACM.0000000000001578 28177958

[ref-7] Burgess A van Diggele C Roberts C : Feedback in the clinical setting. *BMC Med Educ.* 2020;20(Suppl 2): 460. 10.1186/s12909-020-02280-5 33272265 PMC7712594

[ref-8] Carr S : The Foundation Programme assessment tools: an opportunity to enhance feedback to trainees? *Postgrad Med J.* 2006;82(971):576–579. 10.1136/pgmj.2005.042366 16954453 PMC2585733

[ref-9] Castleberry A Nolen A : Thematic analysis of qualitative research data: is it as easy as it sounds? *Curr Pharm Teach Learn.* 2018;10(6):807–815. 10.1016/j.cptl.2018.03.019 30025784

[ref-10] Chary M Parikh S Manini AF : A review of natural language processing in medical education. *West J Emerg Med.* 2019;20(1):78. 10.5811/westjem.2018.11.39725 30643605 PMC6324711

[ref-11] Cushing A Abbott S Lothian D : Peer feedback as an aid to learning - What do we want? Feedback. When do we want it? Now! *Med Teach.* 2011;33(2):e105–e112. 10.3109/0142159X.2011.542522 21275532

[ref-12] Ende J : Feedback in clinical medical education. *JAMA.* 1983;250(6):777–781. 10.1001/jama.250.6.777 6876333

[ref-13] Ferreira-Mello R AndrÉ M Pinheiro A : Text mining in education. *Wiley Interdiscip Rev: Data Min Knowl Discov.* 2019;9(6): e1332. 10.1002/widm.1332

[ref-14] Gigante J Dell M Sharkey A : Getting beyond "good job": how to give effective feedback. *Pediatrics.* 2011;127(2):205–207. 10.1542/peds.2010-3351 21242222

[ref-15] Gilkes L Kealley N Frayne J : Teaching and assessment of clinical diagnostic reasoning in medical students. *Med Teach.* 2022;44(6):650–656. 10.1080/0142159X.2021.2017869 35041564

[ref-16] Gupta K Badyal D Mahajan R : Introduction of structured feedback to medical undergraduate students in the first professional. *Int J Appl Basic Med Res.* 2021;11(1):21–26. 10.4103/ijabmr.IJABMR_138_20 33842291 PMC8025954

[ref-17] Haffling AC Beckman A Edgren G : Structured feedback to undergraduate medical students: 3 years’ experience of an assessment tool. *Med Teach.* 2011;33(7):e349–e357. 10.3109/0142159X.2011.577466 21696267

[ref-18] Han ER Yeo S Kim MJ : Medical education trends for future physicians in the era of advanced technology and artificial intelligence: an integrative review. *BMC Med Educ.* 2019;19(1):1–15. 10.1186/s12909-019-1891-5 31829208 PMC6907217

[ref-19] Hattie J Timperley H : The power of feedback. *Rev Educ Res.* 2007;77(1):81–112. 10.3102/003465430298487

[ref-20] Hynninen T Knutas A Hujala M : Sentiment analysis of open-ended student feedback. *2020 43rd International Convention on Information, Communication and Electronic Technology (MIPRO)*. IEEE,2020.755–759. 10.23919/MIPRO48935.2020.9245345

[ref-21] John B Narayanan G Al-Sawad M : Assessing clinical skills of nursing students: a triangulation study to explore faculty experiences and feedback in Objective Structured Clinical Examination (OSCE). *World Journal of Nursing Research.* 2021. 10.31586/wjnr.2021.105

[ref-22] Khanbhai M Anyadi P SYMONS J : Applying natural language processing and machine learning techniques to patient experience feedback: a systematic review. *BMJ Health Care Inform.* 2021;28(1): e100262. 10.1136/bmjhci-2020-100262 33653690 PMC7929894

[ref-23] Kumar SN : World towards advance web mining: a review. *American Journal of Systems and Software.* 2015;3(2):44–61. Reference Source

[ref-24] Lai MM Roberts N Mohebbi M : A randomised controlled trial of feedback to improve patient satisfaction and consultation skills in medical students. *BMC Med Educ.* 2020;20(1): 277. 10.1186/s12909-020-02171-9 32819352 PMC7439652

[ref-25] Li J Shin SY Lee HC : Text mining and visualization of papers reviews using R language. *J Inf Commun Converg.* 2017;15(3):170–174. 10.6109/jicce.2017.15.3.170

[ref-26] Maimone C Dolan BM Green MM : Utilizing natural language processing of narrative feedback to develop a predictive model of pre-clerkship performance: lessons learned. *Perspect Med Educ.* 2023;12(1):141–148. 10.5334/pme.40 37151853 PMC10162355

[ref-27] Sharma N Jain V : Evaluation and summarization of student feedback using sentiment analysis. *Advanced Machine Learning Technologies and Applications: Proceedings of AMLTA 2020*, Springer,2021. Springer,385–396. 10.1007/978-981-15-3383-9_35

[ref-28] Shrivastava S Shrivastava P : Improving the feedback process in medical education. *Pharmacology.* 2020a;48(1):S5–S9. 10.4038/seajme.v14i1.238

[ref-29] Shrivastava SR Shrivastava PS : Feedback in medical education: changing concepts. *Libyan Journal of Medical Sciences.* 2020b;4(1):3–4. 10.4103/LJMS.LJMS_58_19

[ref-30] Wardman MJ Yorke VC Hallam JL : Evaluation of a multi-methods approach to the collection and dissemination of feedback on OSCE performance in dental education. *Eur J Dent Educ.* 2018;22(2):e203–e211. 10.1111/eje.12273 28524327

[ref-31] Welbers K Van Atteveldt W Benoit K : Text analysis in R. *Commun Methods Meas.* 2017;11(4):245–265. 10.1080/19312458.2017.1387238

[ref-32] Zhang W Cai M Lee HJ : AI in medical education: global situation, effects and challenges. *Education and Information Technologies.* 2023;1–23. 10.1007/s10639-023-12009-8

[ref-33] Zong C Xia R Zhang J : Text data mining. Springer,2021. 10.1007/978-981-16-0100-2

