# Supplementary material for: A retrospective feedback analysis of objective structured clinical examination performance of undergraduate medical students
Source: MedEdPublish (2016). 2024 Oct 24;14:251. [Version 1] doi: 10.12688/mep.20456.1 (PMC11615435; doi:10.12688/mep.20456.1)
Supplement: Peer review history

# Reviewer report: Reviewer response for version 1

**DOI:** 10.21956/mep.21892.r40107

**Published:** 2024-12-05

**Authors:** Ali Al-dabbagh

**Affiliations:** 1 Hawler Medical University, Erbil, Iraq

The authors address an essential aspect of OSCE feedback. They discuss text analysis in detail in the introduction and methods, although some of it is unnecessary.

 The study aims to evaluate and improve the quality of written feedback, but quite obviously, the only thing that was done was using text analysis to show the most frequently used word which was a "good" and this is somewhat expected! in a given cohort of students would expect a bell distribution of grades so a large number of students would receive a "good" in the feedback.

 The authors had access to the whole data of the OSCEs including the grades and global ratings, it would be of benefit if they did a text analysis of those who did not perform well in the exams.

 Based on the result obtained, I don't think the authors can neither conclude that text analysis is useful in this situation nor that implementing structured feedback will improve students learning.

Have any limitations of the research been acknowledged?

Partly

Is the work clearly and accurately presented and does it cite the current literature?

Yes

If applicable, is the statistical analysis and its interpretation appropriate?

Not applicable

Are all the source data underlying the results available to ensure full reproducibility?

Yes

Is the study design appropriate and is the work technically sound?

Partly

Are the conclusions drawn adequately supported by the results?

Partly

Are sufficient details of methods and analysis provided to allow replication by others?

Partly

Reviewer Expertise:

Medical Education, Bioethics, Surgery

I confirm that I have read this submission and believe that I have an appropriate level of expertise to state that I do not consider it to be of an acceptable scientific standard, for reasons outlined above.

# Reviewer report: Reviewer response for version 1

**DOI:** 10.21956/mep.21892.r40118

**Published:** 2024-12-03

**Authors:** Maimoona Nasreen, Kinza Aslam

**Affiliations:** 1 University College of Medicine & Dentistry (UCMD), The University of Lahore (UOL), Lahore, Pakistan

A very important topic has been addressed by the authors. Feedback in OSCEs facilitates self-reflection and helps students become competent in clinical skills. One suggestion to strengthen the research is to identify the patterns that are specific to different stations over time. This will allow for an in-depth exploration of potential areas for improvement and effective feedback.

Have any limitations of the research been acknowledged?

Yes

Is the work clearly and accurately presented and does it cite the current literature?

Yes

If applicable, is the statistical analysis and its interpretation appropriate?

Not applicable

Are all the source data underlying the results available to ensure full reproducibility?

Yes

Is the study design appropriate and is the work technically sound?

Yes

Are the conclusions drawn adequately supported by the results?

Yes

Are sufficient details of methods and analysis provided to allow replication by others?

Yes

Reviewer Expertise:

NA

We confirm that we have read this submission and believe that we have an appropriate level of expertise to confirm that it is of an acceptable scientific standard.

# Reviewer report: Reviewer response for version 1

**DOI:** 10.21956/mep.21892.r40114

**Published:** 2024-11-25

**Authors:** Lamia Yusuf

**Affiliations:** 1 Medical education, consultant medical educationist, University of Health sciences Lahore, Lahore, Punjab, Pakistan

The study focuses on the importance of feedback in, (OSCEs) over five years. It was ethically approved by the University Ethics Committee of the University of Galway, and a waiver of consent was granted due to its retrospective nature. A retrospective analysis of 1034 Written feedback data was analyzed using R software to identify frequently repeated words, visualized through word clouds. The word clouds revealed that the most frequently repeated word in feedback over five years was "good. This suggested a structured feedback form. The study was ethically approved by the University Ethics Committee of the University of Galway, and a waiver of consent was granted due to its retrospective nature. This study addresses a significant aspect of learning: feedback and the need for a structured feedback form. Although limited to one study centre, such studies should involve more centres to increase generalizability. also, studies should involve more clinical years, so a longitudinal study can compare trends and improvements in feedback over some time. New techniques should be used for text-mining analysis.

Have any limitations of the research been acknowledged?

Yes

Is the work clearly and accurately presented and does it cite the current literature?

Yes

If applicable, is the statistical analysis and its interpretation appropriate?

Yes

Are all the source data underlying the results available to ensure full reproducibility?

Yes

Is the study design appropriate and is the work technically sound?

Yes

Are the conclusions drawn adequately supported by the results?

Yes

Are sufficient details of methods and analysis provided to allow replication by others?

Yes

Reviewer Expertise:

medical education, Work place based assessments, simulation-based learning, curriculum, quality assurance in clinical settings.

I confirm that I have read this submission and believe that I have an appropriate level of expertise to confirm that it is of an acceptable scientific standard.

# Reviewer report: Reviewer response for version 1

**DOI:** 10.21956/mep.21892.r40109

**Published:** 2024-11-21

**Authors:** Mohamed Hany Shehata

**Affiliations:** 1 Arabian Gulf University, Manama, Capital Governorate, Bahrain

The study addresses an important topic in medical education – the quality of feedback provided during OSCEs. It used an innovative approach to analyze examiners' written feedback (text mining). The study clearly identifies the dominance of generic terms like "good" and emphasizes the need for more specific feedback. The authors propose practical solutions like structured feedback forms to improve feedback quality.

**The study has several limitations including:**

1. The limited generalizability, as it focuses on a single institution and a subset of students.
2. Data analysis depth as text mining only reveals word frequency, potentially missing the nuances of feedback content.
3. The discussion of AI integration is futuristic and lacks concrete details on its implementation.

This is a well-written that provides valuable insights into the need for more specific feedback in OSCE assessments. However, the authors could strengthen the review by:

**Expand the scope of the study:**

- I am not sure what is the message that can be useful to readers other than the importance of structured feedback. The paper can be enriched by conducting deeper analysis.

**That may consider:**

1. Use topic modeling to identify underlying themes in the feedback to know more about the areas which were of concern to raters,
2. Conduct the analysis for some stations that were used over several years to identify station-specific patterns,
3. Compare feedback from different academic years to identify trends in feedback quality over time.
4. Employ more advanced text mining techniques, such as sentiment analysis, to identify positive and negative feedback.

The discussion part can also be strengthened by discussing the potential impact of generic feedback on student learning and performance, exploring the barriers to providing specific feedback and propose strategies to overcome these challenges, discussing the ethical implications of using AI in feedback provision, such as data privacy and bias.

Have any limitations of the research been acknowledged?

Yes

Is the work clearly and accurately presented and does it cite the current literature?

Yes

If applicable, is the statistical analysis and its interpretation appropriate?

Not applicable

Are all the source data underlying the results available to ensure full reproducibility?

Yes

Is the study design appropriate and is the work technically sound?

Partly

Are the conclusions drawn adequately supported by the results?

Yes

Are sufficient details of methods and analysis provided to allow replication by others?

Yes

Reviewer Expertise:

conduction of OSCE, assessment in medical education, online OSCE

I confirm that I have read this submission and believe that I have an appropriate level of expertise to confirm that it is of an acceptable scientific standard, however I have significant reservations, as outlined above.

## References

[1] : A retrospective feedback analysis of objective structured clinical examination performance of undergraduate medical students. *MedEdPublish* .2024;14: 10.12688/mep.20456.1 10.12688/mep.20456.1 PMC1161543539635542
